# Tunable Particle Focusing in a Straight Channel with Symmetric Semicircle Obstacle Arrays Using Electrophoresis-Modified Inertial Effects

**DOI:** 10.3390/mi7110195

**Published:** 2016-11-01

**Authors:** Dan Yuan, Chao Pan, Jun Zhang, Sheng Yan, Qianbin Zhao, Gursel Alici, Weihua Li

**Affiliations:** 1 School of Mechanical, Materials and Mechatronic Engineering, University of Wollongong, Wollongong, NSW 2522, Australia; dy983@uowmail.edu.au (D.Y.); cp128@uowmail.edu.au (C.P.); jz218@uowmail.edu.au (J.Z.); sy034@uowmail.edu.au (S.Y.); qz260@uowmail.edu.au (Q.Z.); gursel@uow.edu.au (G.A.); 2 School of Mechanical Engineering, Nanjing University of Science and Technology, Nanjing 210094, China

**Keywords:** particle focusing, electrophoresis force, Saffman lift force

## Abstract

In this work, a novel microfluidic platform for tunable particle focusing in a straight channel with symmetric semicircle obstacle arrays using electrophoresis (EP)-modified inertial effects was presented. By exerting an EP force on the charged microparticles, a relative velocity gap between microspheres and fluid in a straight channel with symmetric semicircle obstacle arrays was implemented. The relative velocity and fluid shear will induce shear-slip lift force (Saffman lift force) perpendicular to the mainstream direction. Therefore, the focusing pattern can be altered using the electrophoresis-induced Saffman force. The effects of electric field direction, flow rate, electric field magnitude, and particle size were also studied. This demonstrates the possibility of adjusting the particle inertial focusing pattern in a straight channel with with symmetric semicircle obstacle arrays using electrophoresis. Manipulation of the lateral migration of focusing streaks increases controllability in applications such as blood cell filtration and the separation of cells by size.

## 1. Introduction

Within the last decade, microfluidics has been considered as a technology that provides a miniaturization, integration, automation, and parallelization platform for the application of biomedicine and chemistry, called Lab-on-a-chip, or micro total analysis systems (μTAS) [1]. Compared with the traditional macro-scale platforms (e.g., petri dish, centrifuge, and flow cytometry, etc.), microfluidics has advantages, including fast processing, low cost, high sensitivity, and portability [2,3].

Generally, microfluidics manipulation technologies can be sorted as active and passive, according to the source of the manipulating force. Active techniques such as dielectrophoresis (DEP) [4], magnetophoresis [5], and acoustophoresis [6], etc. employ an external force field to achieve the function; however, passive technologies rely on hydrodynamic forces or channel geometry, such as pinched flow fractionation [7], deterministic lateral displacement [8], and inertial microfluidics [9], etc.

In recent years, inertia-based methods have become extremely popular due to their advantages of simplicity, passiveness, preciseness, continuity, and high-throughput [9,10,11]. Two common channel structures, spiral and serpentine microchannels, have been investigated intensively, and have been widely reported on [12,13,14,15,16,17,18,19]. Recently, some reports have used obstacle structures patterned along one or two sides of the straight microchannel to focus and separate microparticles [20,21,22,23,24]. Recently, the observation that a particle can migrate toward the tube axis when it lags behind the fluid flow has been reported [25]. In a vertical upward Poiseuille flow in a tube, a particle heavier than the fluid lags behind the fluid, which creates a lift force that induces the particle’s migration toward the tube center. This phenomenon was first demonstrated in macroscale flows in a tube [25], and in a rectangular channel [26]. After that, this phenomenon was reported in conjunction with microscale flows, which are intrinsically laminar flows with a Poiseuille parabolic velocity profile [27]. A lagging velocity of particles was induced by utilizing the negative electrophoretic mobility of the charged particles subject to Direct Current (DC) electric field in combination with pressure-driven flow. Therefore, axisymmetric particle focusing can be achieved.

In this work, a novel microfluidic platform for tunable particle focusing in a straight channel with symmetric semicircle obstacle arrays using electrophoresis (EP)-modified inertial effects is presented. Because almost all of the particles and cells will be exerted with net charges on the surface within an aqueous solution, an external direct current electric field can pull or push particles along the main flow stream due to the electrophoretic force, creating relative velocity between the microparticle and the surrounding fluid. The relative velocity and fluid shear will induce shear-slip lift force (Saffman lift force) perpendicular to the mainstream direction. Therefore, the inertial focusing pattern can be adjusted (either to the centre of the channel or to the channel walls) using the external electric field. The particle focusing and migration phenomenon was demonstrated and analysed in this paper by the electrophoresis-modified inertial method. The effects of electric field direction, flow rates, electric field magnitude, and particle sizes were also studied. This demonstrates the possibility of adjusting the particle inertial focusing pattern in a straight channel with symmetric semicircle obstacle arrays using electrophoresis, which may provide high controllability and versatile particle filtration and manipulation platforms for the practical application of biological sample treatment and clinical blood cell filtration.

## 2. Theoretical Background

### 2.1. Inertial Lift Force

In Newtonian fluids, the shear gradient lift force and wall lift force are the two dominant forces governing particle migration, and equilibrium positions are created by the balance of the two lift forces. The sum of the two inertial lift forces (called the net inertial lift force) can be expressed as [9,28]:

(1) $$F_{L}=\frac{\rho_{f}U_{m}^{2}a^{4}}{D_{h}^{2}}f_{L}(R_{c},x_{c})$$

(2) $$R_{c}=\frac{\rho_{f}U_{m}D_{h}}{\mu_{f}}=\frac{2\rho_{f}Q}{\mu_{f}\left(w+h\right)}$$

where $\rho_{f}$, $U_{m}$, and $\mu_{f}$ are the fluid density, mean velocity, and dynamic viscosity, respectively; *a* is the spherical diameter of the particles; $D_{h}=2wh/(w+h)$ is the hydraulic diameter of a rectangular channel, with w and h the width and height of the channel cross-section. *Q* is the flow rate. The lift coefficient of net inertial lift force $f_{L}(R_{c},x_{c})$ is a function of the position of the particles within the cross-section of channel $x_{c}$ and the channel Reynolds number $R_{c}$ [9].

### 2.2. Electrophoresis

In a spatially-uniform electric field, dispersed particles will move in the fluid in this electric field, and this motion is called electrophoresis [29,30]. According to the famous Helmholtz–Smoluchowski result, the electrophoretic velocity ${\vec{v}}_{EP}$ (with respect to the fluid) is given as follows when Debye lengths are much smaller than particle size [31]:

(3) $${\vec{\upsilon }}_{EP}=\frac{\varepsilon \zeta }{\mu_{f}}\vec{E}$$

${\vec{v}}_{EP}$ is independent of particle size and shape. The permittivity of the fluid is defined by ε. $\mu_{f}$ is dynamic viscosity, and ζ is the ζ-potential [31,32] of the electric double layer surrounding the particle. The electrophoretic mobility is defined by $u_{EP}=\frac{\varepsilon \zeta }{\mu_{f}}$, which is the proportionality factor between particle velocity and electric field $\vec{E}$. The electric field induces the electrophoretic motion; however, $u_{EP}$ is only dependent on the particle charge (hidden in the ζ-potential [31]), and depends on properties of the electric double layer around the particle and the electrolyte.

### 2.3. Dielectrophoresis

In a non-uniform electric field, a net force (known as a DEP force) will exert on the particle. This is because the Coulomb forces on either side of the dipole moment can be different. A DEP motion is that where a particle moves towards or against the region of electric field maxima. The motion is directed by DEP force, which depends on the relative polarizabilities of the particle and the suspending medium [33].

The effective dipole moment of a spherical particle is [34,35]:

(4) $$P=4\pi \varepsilon_{m}r^{3}Re\left[K\left(\omega \right)\right]E$$

where P is a vector, and the absolute permittivity of the suspending medium is defined by $\varepsilon_{m}$. r, K(ω), and Re indicate the particle radius, the Clausius–Mossotti (CM) factor, and the real part. The Clausius–Mossotti (CM) factor depends on the complex permittivity of the particle and the suspending medium, and also the frequency of the external electric field:

(5) $$K\left(\omega \right)=\frac{\varepsilon_{p}^{*}\left(\omega \right)-\varepsilon_{m}^{*}\left(\omega \right)}{\varepsilon_{p}^{*}\left(\omega \right)+2\varepsilon_{m}^{*}\left(\omega \right)}$$

Complex permittivity is defined by $\varepsilon^{*}\left(\omega \right)=\varepsilon -i\sigma /\omega$, where σ is the electrical conductivity ω is the frequency of the electric field. The particle and the suspending medium are described by subscripts *p* and *m*. In the presence of an electric field, the particles and suspending medium both exhibit dielectric and conductive properties, so the complex permittivities are presented here.

### 2.4. Saffman Force (Shear-Induced Lift Force)

Saffman force was recognized as arising from the interaction of the Stokeslet velocity field around the particle with the velocity gradient (shear rate). Using a matched asymptotic expansion method, Saffman [36] calculated the lateral lift force on a sphere in an unbounded simple shear flow, which has constant shear rate and zero shear gradients. The magnitude of this force is

(6) $$F_{S}=\frac{K}{4}Va^{2}{\left(\gamma v^{-1}\right)}^{1/2}$$

where *K* is a numerical constant (*K* ~ 81.2), velocity gradient or shear rate is defined by γ, *V* is the relative velocity (velocity difference between fluid velocity at the streamline through the center of the particle and the particle), and *v* is kinematic viscosity.

When non-neutrally buoyant particles flow in a vertical fluid, or external force fields (electrical or magnetic) [11,27] induce particles to significantly lag or lead fluid flow, the Saffman force is more relevant, and net Stokeslet flow is more obvious. In this case, the Saffman force will direct to the channel centreline when particles lag the flow and particles migrate to the channel centreline, or the Saffman force will direct to the channel walls when particles lead the flow, and particles migrate to channel walls.

## 3. Materials and Methods

### 3.1. Design and Fabrication of a Microfluidic Device

The microfluidic device was designed with one long straight microchannel with semicircular obstacle arrays, one inlet, one outlet, and two electrode chambers using AutoCAD. The channel consists of a 10 mm straight section with 20 periods of symmetric semicircle obstacles patterned on both sides of a microchannel with a constant distance of 500 μm. The depth of the channel is uniform at 40 μm. The width of the straight microchannel changes is 250 μm, and the dimeter of the semicircular obstacle is 200 μm. The distance of the two electrode chambers is 13 mm. 

The device was fabricated using standard photolithography and soft lithographic techniques [37,38]. This fabrication included rapid prototyping on a silicon master, and polydimethylsiloxane (PDMS) replica molding and sealing through plasma oxidation. 

### 3.2. Particle Preparation

Particle suspensions were prepared by diluting 5 μm internally green dyed fluorescent polystyrene microspheres (coefficient of variation (CV) < 5%, ThermoFisher Scientific, Waltham, MA, USA), and 13 μm internally red dyed fluorescent polystyrene microspheres (CV < 5%, Thermo Fisher Scientific) in deionized (DI) water containing 0.01% (*v*/*v*) Tween 20 (Sigma-Aldrich, San Antonio, TX, USA). Tween 20 was included in suspension to prevent particle aggregation. Before commencing each experiment, the particle solutions were re-suspended by vortex to provide uniform suspensions. 

### 3.3. Experimental Setup

**Figure 1:**
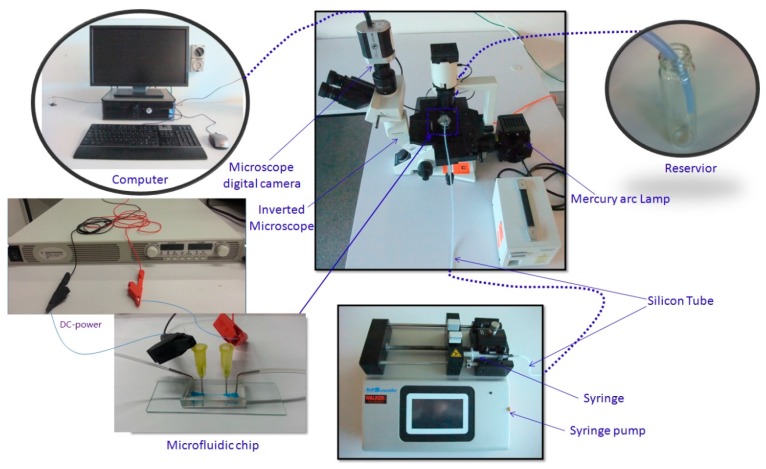
Experimental setup.

The microfluidic device was placed on an inverted microscope (CKX41, Olympus, Japan), illuminated by a mercury arc lamp. The particle suspension was infused into the microchannel with specific flow rate by a programmable syringe pump (Legato 100, Kd Scientific, Holliston, MA, USA). The electric field was generated by a DC-electric source (N5772A, Agilent Technologies, Santa Clara, CA, USA). The fluorescence images were observed and captured by a CCD camera (Rolera Bolt, Q-imaging, Albion, Australia) and then post-processed by Q-Capture Pro 7 software (Q-imaging). The exposure time for each frame was set at 100 ms. The experimental setup is shown in Figure 1. 

## 4. Results and Discussion

### 4.1. Schematic of Tunable Particle Focusing in the Straight Channel with Symmetric Semicircle Obstacle Arrays Using EP-Modified Inertial Effects

A non-uniform electric field can be generated within this channel when a DC power is imposed near the inlet and outlet of the channel. The particles experience a dielectrophoretic force (*F*_DEP_) induced by the non-uniform electric field. Meanwhile, because the polystyrene particles are negatively charged in the electrolyte solution [27], they experience an electrophoretic force (*F*_EP_) as well. The direction of the electrophoretic force is opposite to the direction of electric field. The electrophoretic force can decrease or increase the particle speed to induce particles to lag behind or lead the flow. The relative velocity and fluid shear can induce Saffman lift force perpendicular to the mainstream direction, which can drive particles to migrate toward the channel center or to the channel walls.

**Figure 2:**
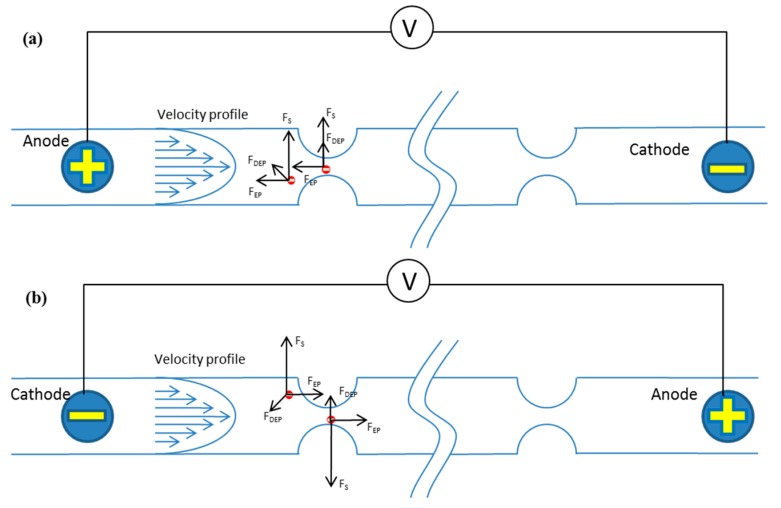
Schematic of tunable particle focusing in a straight channel with symmetric semicircle obstacle arrays using electrophoresis (EP)-modified inertial effects. (**a**) The principle of radially-inward (toward the channel center) migration of particles by the electrophoresis-induced Saffman force; (**b**) The principle of radially-outward (toward the channel wall) migration of particles by the electrophoresis-induced Saffman force.

The schematic of tunable particle focusing in a straight channel with symmetric semicircle obstacle arrays using EP-modified inertial effects is shown in Figure 2. The direction of the electric field is reversed in Figure 2a,b. In Figure 2a, the anode is near the inlet, while the cathode is near the outlet, and the electric field direction is from inlet to outlet; in Figure 2b, the cathode is near the inlet, the anode is near the outlet, and the electric field direction is from outlet to inlet. Figure 2a,b show the principle of radially-inward (toward the channel center) and radially-outward (toward the channel wall) migration of particles by the electrophoresis-induced Saffman force, respectively. In this channel with the DC-electric field, particles are actually affected by dielectrophoretic, inertial, electrophoretic, and Saffman lift forces. However, the inertial lift force can be neglected when the flow rate is very low, and the dielectrophoretic force is too weak compared to electrophoretic and Saffman lift force. The schematic figure only demonstrates the additional forces (dielectrophoretic, electrophoretic, and Saffman lift forces) on the basis of inertial focusing. When the electrophoretic force direction is inverse to the flow direction of particles, the particles will lag behind the flowing fluid; therefore, the Saffman lift force directs to the center of the channel, and particles migrate to the channel centerline (Figure 2a). When the electrophoretic force direction is the same as the fluid flow direction exerted by the external pressure, the particles will lead the flowing fluid; therefore, the Saffman lift force directs to the channel sidewalls, and particles are modified to migrate toward channel walls (Figure 2b).

### 4.2. Effects of Electric Field Direction

**Figure 3:**
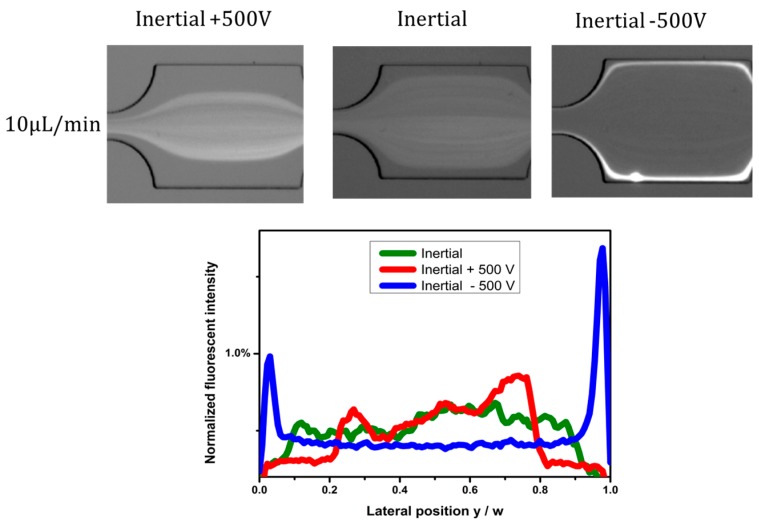
Effects of electric field direction.

Experiments were carried out by inserting an anode near the inlet, and a cathode near the outlet, while pumping fluid containing 5 µm particles into the channel. The electric field is from the inlet to outlet, and its direction was reversed by reversing the anode and cathode. Figure 3 shows the effects of electric field direction. The particle distribution under pure inertial flow conditions is used for comparison (Figure 3, Inertial). In Figure 3 (left, Inertial +500 V), the direction of electrophoretic force is inverse to the flow direction of the particles, and the particles lag behind the flowing fluid; therefore, the Saffman lift force directs toward the center of the channel, and particles migrate to channel centerline under the combination of dielectrophoretic force (*F*_DEP_), electrophoretic force (*F*_EP_), and Saffman force (*F*_S_). Compared with the particle distribution under the pure inertial condition, the particle focusing width is much narrower under radially-inward Saffman force. When the direction of the electric field is from outlet to inlet, the direction of the electrophoretic force imposed on the negatively-charged particles is the same as the flow direction, and then the particles lead the flowing fluid; therefore, the Saffman lift force directs to the channel sidewalls, and particles migrate to channel walls under the combination of forces (Figure 3, Inertial −500 V). Compared with the particle distribution under the pure inertial conditions, the particles were pushed to the channel walls under radially-outward Saffman force. As can be seen from the corresponding fluorescent profiles as well, the particles’ focusing width becomes narrower by radially-inward Saffman force, and particles are focused near channel walls under radially-outward Saffman force. 

### 4.3. Effects of Flow Rates

**Figure 4:**
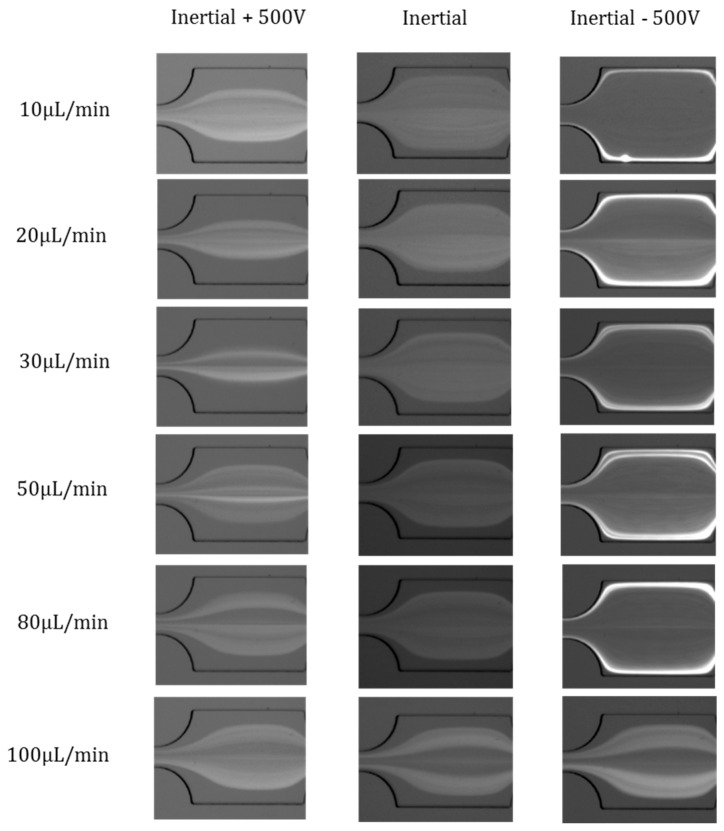
Effects of flow rates on particle distribution in Inertial +500 V, pure Inertial, and Inertial −500 V cases.

The effects of flow rates were investigated at fixed electric voltages of +500 V and −500 V, respectively. The flow rate range is from *Q* = 10 µL/min to *Q* = 100 µL/min. The particle distribution under pure inertial flow conditions is demonstrated for comparison (Figure 4, Inertial). In the inertial +500 V case, the Saffman force is radially-inward, and the particles’ focusing width becomes narrower by Saffman force. When the flow rate is relatively low (from *Q* = 10 µL/min to *Q* = 30 µL/min), the inertial effects can be neglected. Therefore, the Saffman force can overcome the inertial lift force and push particles toward the center of the channel. However, as the flow rate increases (*Q* = 50 µL/min to *Q* = 100 µL/min), the inertial effect begins to become dominant, thus there is no significant difference in the distribution of particles at pure Inertial and Inertial +500 V. In the Inertial −500 V case, the Saffman force is radially-outward, and the particles are pushed to the channel walls by Saffman force. When the flow rate is relatively low (from *Q* = 10 µL/min to *Q* = 80 µL/min), the inertial effects are very weak. Therefore, the Saffman force can overcome the inertial lift force and push particles toward the channel walls. However, as the flow rate increases further (*Q* = 100 µL/min), the inertial effect becomes dominant, and the Saffman force cannot overcome the inertial effects; thus, there is little difference in particle distribution at pure Inertial and Inertial −500 V. We also found that the effects of the radially-outward Saffman force occupy a much wider flow rate range than that of the radially-inward Saffman force. This is because the fluid flow and particle velocities near channel walls are much slower than that at the channel centre, and so a weak Saffman force can migrate particles toward sidewalls.

### 4.4. Effects of Electric Field Magnitude

**Figure 5:**
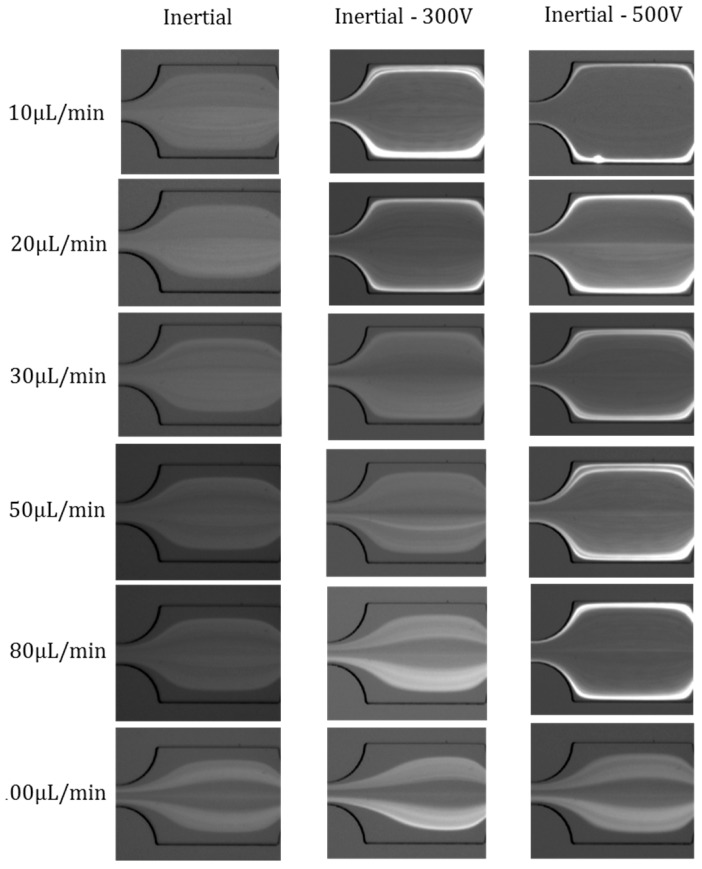
Effects of electric field magnitude on particle distribution (Inertial, Inertial −300 V, Inertial −500 V) from flow rate 10 µL/min to 100 µL/min. The electric field direction is from the outlet to the inlet.

The Saffman force can induce radially-inward or radially-outward particle migration, according to the direction of electric field; meanwhile, the magnitude of the electric field can also influence the particle distribution. The particle distributions are compared at different voltages (Inertial −300 V, Inertial −500 V) at each flow rate from 10 µL/min to 100 µL/min (Figure 5). When the voltage is 0 V, or under pure inertial condition, the particles are randomly distributed; once the voltages are imposed, the particles velocity leads the flowing fluid, therefore, the Saffman lift force directs to the channel sidewalls, and particles aggregate along the channel walls. The radially-outward particle migration is more obvious in Inertial −500 V compared with that in Inertial −300 V circumstance. However, when the flow rate increases to a certain value (30 µL/min), the inertial effect cannot be neglected; thus, the Saffman lift force in a relatively lower electric field cannot overcome the inertial effect (see Inertial −300 V), while the radially-outward particle migration is still obvious when *Q* = 80 µL/min in the Inertial −500 V case. At a flow rate of 100 µL/min, inertial effects overcome the Saffman force; thus, particle distributions are almost the same in Inertial, Inertial −300 V, and Inertial −500 V circumstances. In summary, the Saffman force is more dominant in larger electric fields and slower flow rates.

### 4.5. Effects of Particle Size

**Figure 6:**
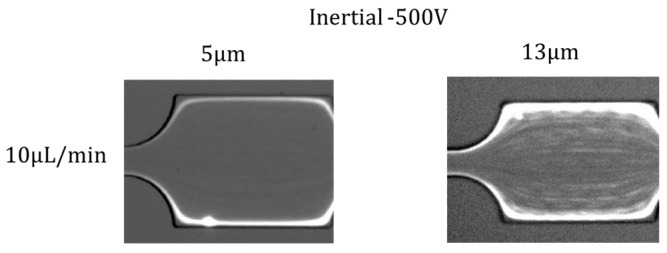

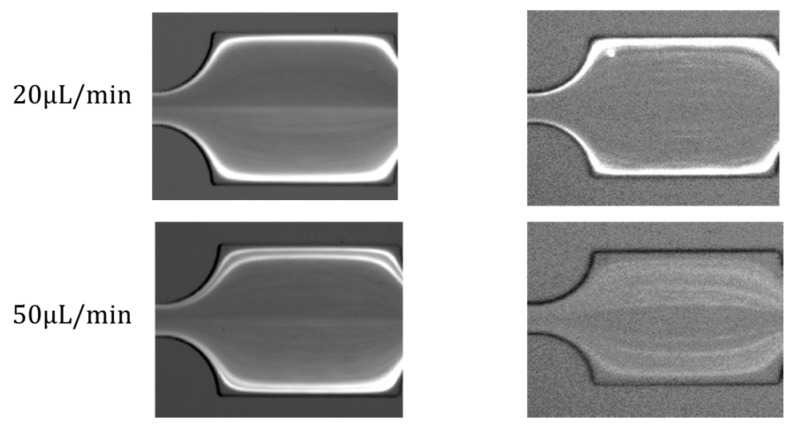
Distribution of 5 µm and 13 µm particles in Inertial −500 V at flow rate 10 µL/min, 20 µL/min, and 50 µL/min.

In addition, we investigated the effects of particle size on particle distribution under the effects of Saffman force. The distribution of 5 µm and 13 µm particles in Inertial −500 V at flow rate 10 µL/min, 20 µL/min, and 50 µL/min is shown in Figure 6. Compared with 5 µm particle distribution, 13 µm particles are more randomly distributed at the channel central area, although two obvious focusing lines along the channel sidewalls can be observed. This is because, for particles with larger size, the inertial effect is more obvious, and particles are prone to be focused at the center of the channel (the inertial lift force is proportional to the fourth power of the particle size, while the Saffman force is proportional to the second power of the particle size). However, the saffman force cannot entirely overcome inertial lift force, and there is still a large amount of larger particles distributed across the whole channel. The inertial effect becomes more dominant at a higher flow rate; therefore, this phenomenon becomes more obvious as the flow rate increases (50 µL/min). Meanwhile, the inertial effects of smaller particles are much weaker than that of the larger particle; therefore, Saffman force can still pinch particles along two sidewalls when the flow rate is increased to 50 µL/min. In summary, the Saffman force is more dominant for particles with smaller sizes.

## 5. Conclusions

In summary, a novel microfluidic platform for tunable particle focusing in a straight channel with symmetric semicircle obstacle arrays using EP-modified inertial effects was presented. By exerting an EP force on the charged microparticles, the focusing pattern can be adjusted using the electrophoresis-induced Saffman force. Compared with particle distribution in pure inertial condition, particles’ focusing width becomes narrower by radially-inward Saffman force, and particles are pushed to the channel sidewalls under radially-outward Saffman force. Based on this phenomenon, the effects of electric field direction, flow rate, electric field magnitude, and particle size were studied. This demonstrates the possibility of adjusting the particle inertial focusing pattern in a straight channel with symmetric semicircle obstacle arrays using electrophoresis, and this device has the potential to be used in biological or chemical applications, such as filtration, separation by size, or by the surface charge of bioparticles.
